# Encoding innate ability through a genomic bottleneck

**DOI:** 10.1073/pnas.2409160121

**Published:** 2024-09-12

**Authors:** Sergey Shuvaev, Divyansha Lachi, Alexei Koulakov, Anthony Zador

**Affiliations:** ^a^Cold Spring Harbor Laboratory, Cold Spring Harbor, NY 11724

**Keywords:** neural computation, neural networks, machine learning, AI

## Abstract

Animals are born with extensive innate behavioral capabilities, which arise from neural circuits encoded in the genome. However, the information capacity of the genome is orders of magnitude smaller than that needed to specify the connectivity of an arbitrary brain circuit, indicating that the rules encoding circuit formation must fit through a “genomic bottleneck” as they pass from one generation to the next. Here, we formulate the problem of innate behavioral capacity in the context of artificial neural networks in terms of lossy compression of the weight matrix. We find that several standard network architectures can be compressed by several orders of magnitude, yielding pretraining performance that can approach that of the fully trained network. Interestingly, for complex but not for simple test problems, the genomic bottleneck algorithm also captures essential features of the circuit, leading to enhanced transfer learning to novel tasks and datasets. Our results suggest that compressing a neural circuit through the genomic bottleneck serves as a regularizer, enabling evolution to select simple circuits that can be readily adapted to important real-world tasks. The genomic bottleneck also suggests how innate priors can complement conventional approaches to learning in designing algorithms for AI.

## Significance

Our manuscript formulates and provides a solution to a central problem in computing with neural circuits: How can a complex neural circuit, with trillions of individual connections, arise from a comparatively simple genome? What makes this problem challenging is the largely overlooked fact that these circuits, at or soon after birth and with minimal learning, are able to specify a tremendously rich repertoire of innate behaviors. The fact that animals are endowed with such sophisticated and diverse innate behaviors is obvious to anyone who has seen a spider spin a web. We formulate the question in terms of artificial networks, which allows us a rigorous and quantitative framework for assessing our ideas.

Many animals are born with impressive and elaborate behavioral capacities. Soon after birth, a spider can build a web, a whale can swim, and a monkey fears snakes. From an evolutionary perspective, it is easy to see why such innate abilities would be selected for: Those individuals that can survive beyond their most vulnerable early hours, days, or weeks are more likely to survive until reproductive age and hence produce progeny at a higher rate. Of course, in practice, there is no crisp distinction between innate and learned abilities; innate abilities form a foundation for learning, and animal behavior arises from the interaction between these two processes. Although learning has been studied extensively in the context of AI, there has been much less theoretical attention devoted to the structure of innate behaviors.

Innate behaviors are encoded in the genome and can be expressed in the neural circuits already present at birth. However, this poses a challenge: How can a complex neuronal connectivity diagram be encoded into a genome? The size of the genome provides an approximate upper bound on the amount of information transmitted from generation to generation. The genome of a simple worm *Caenorhabditis elegans* is about $10^{8}$ base pairs (1), so it could transmit up to about $2\times 10^{8}$ bits. This in principle would be more than adequate to explicitly encode the highly stereotyped connectivity among the 302 neurons in the *C. elegans* brain, since even a dense $302^{2}$ connection matrix would take at most $9\times 10^{4}$ bits to store, times a small factor associated with the number of bits per synaptic weight. On the other hand, the human genome is only about an order of magnitude larger than that of *C. elegans* (∼10^9^ bits), whereas the human cortex has about $10^{10}$ neurons, so even the (sparse) cortical connectivity matrix might require at least $10^{15}$ bits to specify. This implies that the human cortex would require about 5 to 6 orders of magnitude more information to specify than is available in the genome if every connection were specified explicitly (2). Since the genome encodes the rules for wiring up the nervous system, a natural question is how the small amount of information contained in the genome can instruct the creation of the large-capacity cortex. We refer to the long recognized (3–5) mismatch between the information capacity of the genome and the complexity of the resulting neural circuit as a “genomic bottleneck” (2).

**Fig. 1.**
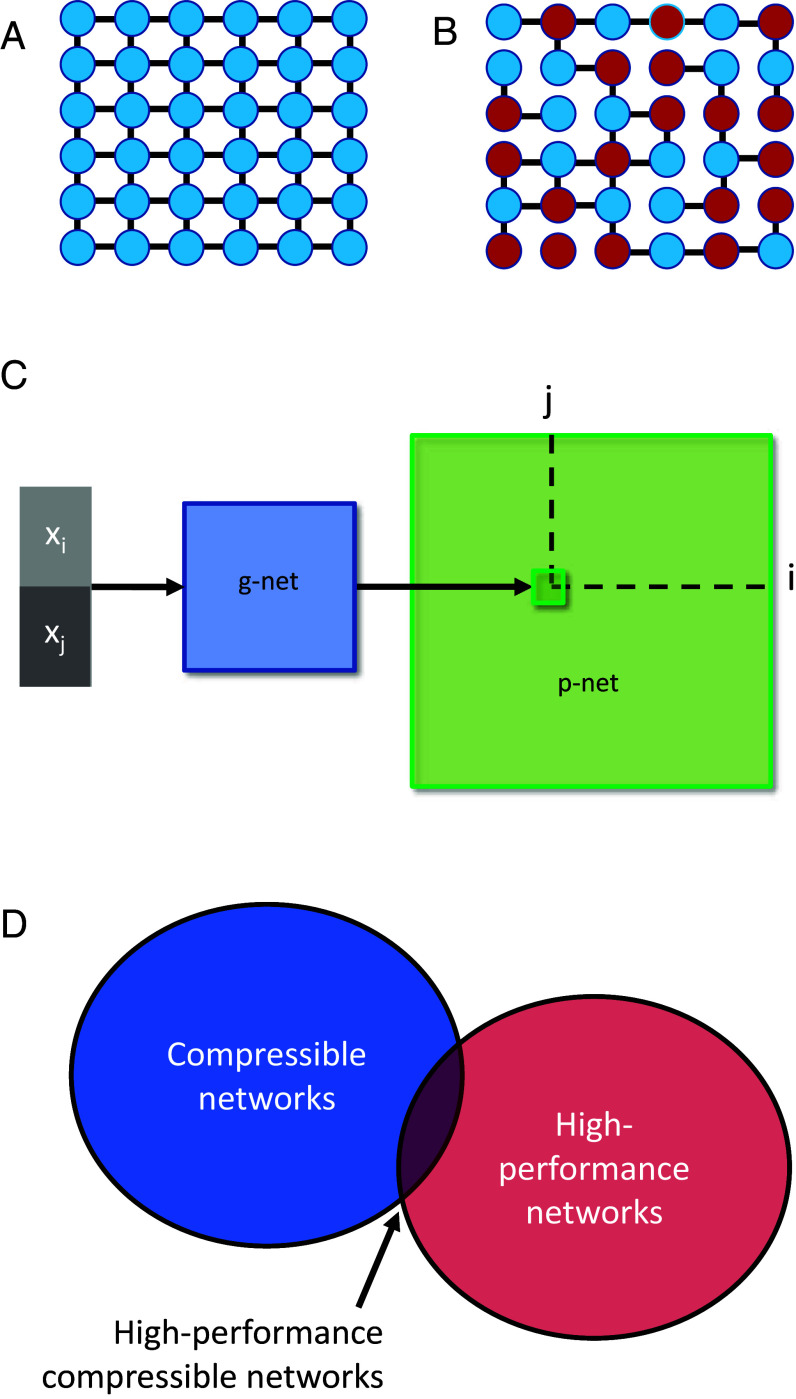
Simple rules can specify networks. (*A*) A very simple nearest-neighbor wiring rule. (*B*) A somewhat more complex rule (“only connect to nearest neighbors of opposite color”) leads to a more complex network. (*C*) Network specification through a genomic bottleneck. The input to the genomic network (“g-network”) is a pair of neurons (pre- and postsynaptic) specified by binary strings. Each neuron has a unique label, consisting of a unique binary string. The two binary labels are passed through the g-network, which assigns the strength of the connection between the two neurons in the “p-network.” Because the number of parameters in the g-network is smaller than the number of parameters in the p-network, the g-network is effectively compressing the p-network. (*D*) G-networks seek to discover p-networks that both solve the problem well and are compressible.

The mismatch described by the genomic bottleneck implies that the connectivity of most neuronal circuits, including the mammalian cortex, is not explicitly specified, neuron-by-neuron, in the genome. Rather, the genome specifies connectivity rules. It has long been recognized that simple rules can give rise to surprisingly complex structures (6, 7), and it is straightforward to formulate simple, low-complexity rules that specify the connectivity in arbitrarily large networks. For example, the simple rule “connect to your four nearest neighbors” specifies a grid of potentially unlimited size (Fig. 1*A*). Another class of rules specifies connections between cells based on the surface markers they express (4, 8, 9) (Fig. 1*B*); axons can exploit these markers to find their destinations (10). The columnar organization that is observed in many brain regions allows for the replication of similar connectivity modules throughout the brain, thus limiting the number of parameters needed to wire the circuit (11). Complex structures such as orientation columns in the visual cortex can be induced to self-organize from simple use-dependent rules (12). Developmental rules such as these can dramatically reduce the amount of information needed to specify the connectivity of a neural circuit, before extensive experience.

Despite this extensive literature on modeling development, these simple developmental rules do not typically specify networks with the capacity to perform complex general computations. Thus, although such rules can readily specify the formation of repeated modules that enable the emergence of receptive fields in the retina or the visual cortex (13), such stereotyped modules cannot directly encode more specialized knowledge like a spider’s capacity to build a web or a rat’s innate fear of fox odor (14). We therefore set out to explore how low-complexity rules can give rise to networks that perform complex well-defined neural computations. Within the framework of artificial neural networks (ANNs), we seek to compress the complex connectivity (weight matrix) into a much smaller “genome.” The decoding of this genome into the initial weights of the network enables the network to perform well upon initialization, without additional training. This decoding is analogous to the neurodevelopmental processes by which the genome provides a blueprint for circuits that enable animals to perform essential tasks at or soon after birth. We hypothesized that under some conditions, compressing the weight matrix through a “genomic bottleneck” would extract the most useful and important features of the connectivity; the genome would act as an “information bottleneck” (15–17). In this way, a physical constraint—the limited size of the genome—might be an algorithmic advantage, serving as a regularizer and thereby turning a potential “bug” into a feature.

## Implementation of the Genomic Bottleneck

To test these ideas, we first trained standard feedforward ANNs on well-studied supervised learning tasks. ANNs consist of nodes (“neurons”) connected by weights (“synapses”). When an ANN learns a task, the “knowledge” of the task is summarized in the weights of the ANN. Artificial neural networks are typically initiated randomly—tabula rasa—and acquire their functionality through learning, although most ANNs [e.g. convolutional neural networks (18)] are endowed with domain-specific inductive priors via their architecture. Here, we use “connectivity” to describe both the specification of which connections are nonzero, as well as the strengths or weights of those connections. We refer to the trained network as the “phenotype network,” or “p-network.”

We sought to compress the p-network through a genomic bottleneck, preserving as much of the performance as possible. The compression mechanism serves to initialize the network, endowing it with innate abilities prior to learning. To search widely over the space of possible compressions, we used a separate ANN—a “genomic network” or “g-network”—to generate the p-network (Fig. 1*C*). The formulation of the neurodevelopmental process as an ANN allows us to focus on the genomic bottleneck at a conceptual level, without the need to model the complexities of neural development. This leads to a model in which genomes and the circuits they encode are co-optimized in nested loops: an inner loop corresponding to “learning” in animals and an outer loop corresponding to “evolution.” For reasons of efficiency, in our model, both the inner and outer loops are optimized by gradient descent and are not intended as detailed models of learning or evolution.

The inputs to the g-network are the identifiers of a pair of pre- and postsynaptic neurons, each represented by a unique binary vector. In these binary vectors, individual digits represent the presence of one type of a “molecular tag” in the expression profile of this neuron. Thus, for example, if each neuron is represented by 10 binary digits, the input layer of the g-network will consist of 20 units (10 each for presynaptic and postsynaptic neurons; see *Methods*). The output of the g-network is the expected strength of the connection between these neurons. The connectivity is effectively guided by the interactions of pairs of molecules expressed on the pre- and postsynaptic membranes. This formulation is inspired by neurodevelopmental rules based on local pair-wise interactions between neurons (4, 8), but it is not intended as a realistic model of neural development (19, 20). To facilitate the efficient search for g-networks that achieve good compression, we used stochastic gradient descent (21) rather than evolutionary algorithms (5, 19) to achieve the optimization of both the g-network and the p-network.

Although a sufficiently large g-network could, in principle, perfectly recapitulate the p-network by “memorizing” all of the connections exactly, this would fail to compress the p-network, and thus would be unlikely to extract more general wiring motifs. We therefore focused on a regime where the size (complexity) of the g-network is substantially smaller than the size of the p-network, encouraging the g-network to discover compact wiring rules. Our goal is to find network architectures that are both high-performance and compressible (Fig. 1*D*). This setting can be viewed as a lossy compression problem, where the success of compression is measured not by, e.g., the reconstruction error as in typical lossy image compression, but rather by the ability of the uncompressed weight matrix to perform well on the target task without further training.

Our approach can be described as the search for a solution that balances two competing goals. First, we wish to achieve a good innate performance, i.e. we wish to minimize the error $E_{0}\left(G\right)$, where $E_{0}\left(G\right)$ is the error of the p-network on a target task before the training of the p-network encoded by the corresponding g-network G. Second, we also wish to limit the complexity of the genomic network, specifically the entropy H(G) of the genome G. We use the number of parameters specifying the g-networks as a surrogate for the entropy H(G). Conceptually, we can thus formulate our overall goal as a lossy compression problem in which we seek to minimize an objective function J with respect to the genome parameters G:

[1] $$\begin{array}{c} J=E_{0}\left(G\right)+\gamma H\left(G\right), \end{array}$$

where γ is a positive parameter that specifies the tradeoff between the two goals. (In practice, we select a particular complexity H(G) and then minimize the error for that network). In this formulation, the second term can be seen as a regularizer, related to techniques such as weight pruning (22, 23), that seek to keep the weight matrix simple.

We train g-networks iteratively to generate compressible and efficient p-networks as follows. First, we initialize a p-network using random weights and train it for several steps by updating its weights $W^{\left(1\right)}$ to reduce the cost function on the p-network’s target task. Throughout the training process, we maintain the domain-specific architecture of the ANN, such as the convolutional or feedforward structure. The p-network’s updated weights $W^{\left(1\right)}$ are then used to train g-networks. A training set for a g-network consists of vectors $\left\{b_{i},b_{j}\right\}$ encoding the {i,j} position in $W^{\left(1\right)}$ as inputs, and scalars $w_{\mathrm{ij}}^{\left(1\right)}$ containing the strengths of corresponding weights as outputs. The g-network generates an approximation ${\hat{W}}^{\left(1\right)}$ of the p-network’s weight matrix $W^{\left(1\right)}$. The cost function of the g-network training is the difference between p-network weights $W^{\left(1\right)}$ and the approximation generated by the g-network ${\hat{W}}^{\left(1\right)}$. This approximation is then used to initialize the p-network’s weights which serve as the initial weights for the second iteration $W^{\left(2\right)}={\hat{W}}^{\left(1\right)}$. The p-network is trained again directly on its target task. The process is iterated, yielding the sequence ${\hat{W}}^{\left(2\right)}\ldots {\hat{W}}^{\left(K\right)}$ that aims to converge at high zero-shot (innate) performance of the p-network while occupying limited space in the genome (stored in g-networks).

## Supervised Learning

### Application to the MNIST Dataset.

**Fig. 2.**
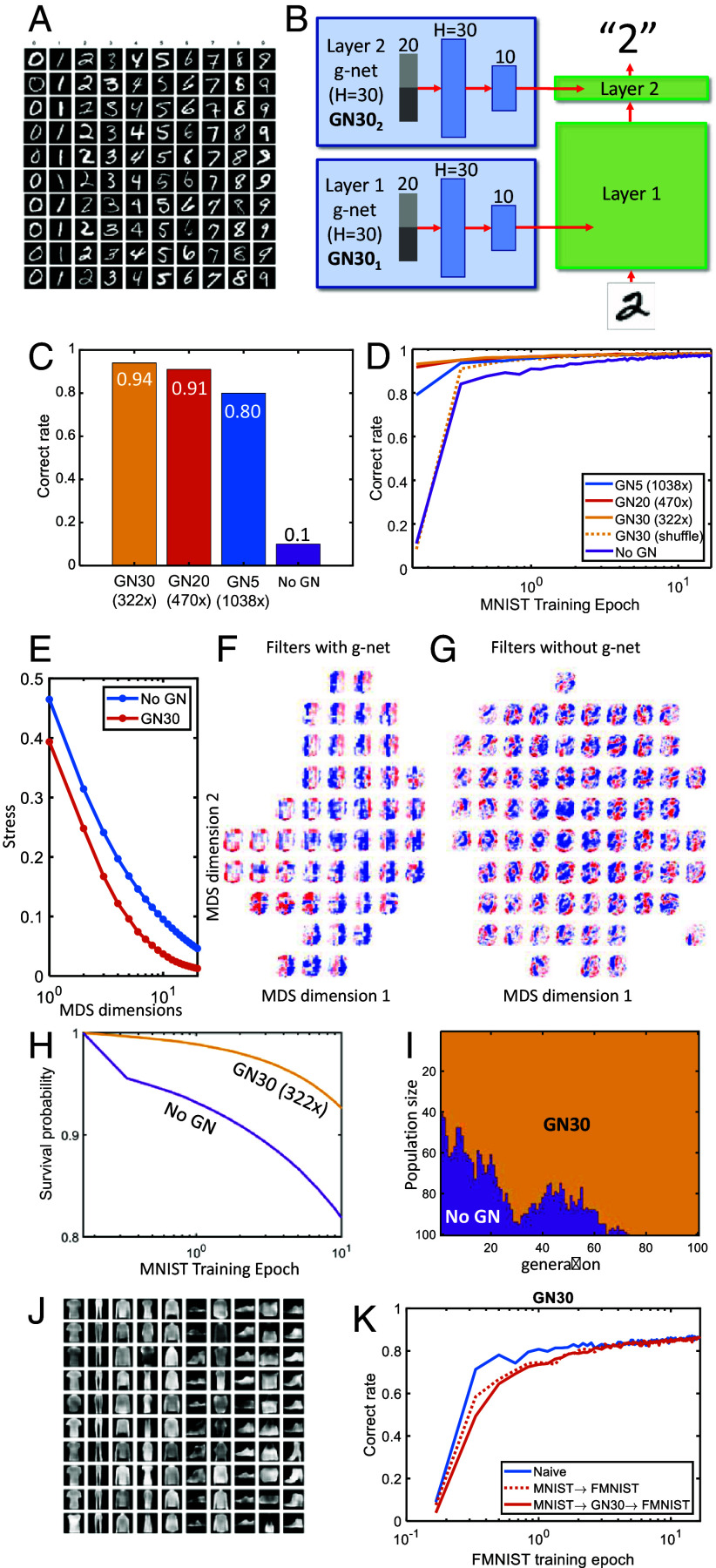
Genomic bottleneck approach to MNIST. (*A*) Examples of handwritten digits from the MNIST dataset. (*B*) We used a two-layer fully connected MNIST network created by individual g-networks for each layer (GN30_1_ denotes a g-network with H=30 units in the hidden layer for the first MNIST layer). GN30 corresponds to 322-fold compression. (*C*) Initial performance for several levels of compression. Here and below, the performance is shown for held-out test data. Performance is excellent even with 1,038-fold compression. (*D*) Training dynamics of p-networks initialized with different levels of compression. The first point for each curve corresponds to the test set accuracy of an untrained p-network initialized by a g-network, as specified. (*E*) The MDS stress for g-network-generated weights (red) and the weights of the network trained without g-network (blue). (*F* and *G*) The average-case filters, applied to pixel space, in (*F*) the g-network-generated weight and (*G*) a weight learned without a g-network. (*H*) The fitness advantage of a model organism with high innate performance. (*I*) A model organism with high innate performance dominates the population. (*J*) Example of the F-MNIST dataset. (*K*) Failure of transfer learning the F-MNIST dataset. Blue, dotted, and solid red lines represent results for training a network using the F-MNIST dataset that is initialized by random, MNIST weights, and weights generated using GN30 trained on the MNIST data respectively.

We first tested our approach on a classic supervised learning problem: handwritten digit recognition. In this problem, a network is trained with examples of handwritten digits (0 to 9) taken from the MNIST dataset (Fig. 2*A*; *Methods*) and learns to assign labels to new examples of these digits it has not previously encountered. For the p-network, we used a standard fully connected network architecture with 28×28=784 pixel units at the input layer and one hidden layer with 800 units, for a total of $6\times 10^{5}$ parameters. With random weights at the initialization, the performance rose during the training from random (10%) to about 98% (Fig. 2*D*) after about 20 epochs.

We then used a much smaller g-network with only 30 hidden units (GN30), or about $2\times 10^{3}$ parameters, to compress the p-network (Fig. 2*B*). To assess the p-network’s performance here and in all subsequent results, we used held-out test data. The g-network trained using the algorithm described above generated a p-network with 94% correct performance upon initialization. Thus the g-network was able to achieve 322-fold compression while maintaining innate performance almost equal to that of the fully trained network. We observed a tradeoff between the degree of compression and the innate performance (Fig. 2 *C* and *D*), but a good innate performance of 79% correct could even be achieved using the GN5, a network with 1,038-fold compression. To test whether the g-net was learning more than the initial distribution of weights (e.g. Xavier), we performed a control experiment in which we shuffled the weights at test time (Fig. 2*D*, dashed yellow line). Initial performance was at chance (0.1), although learning was markedly accelerated. These results demonstrate that there exist p-networks that are both compressible and high-performance.

To explore the effect of the genomic compression on features extracted by the p-network, we applied the multidimensional scaling (MDS), a nonlinear dimensionality reduction technique (24), to the weights in both the compressed and uncompressed networks. Fig. 2*E* shows that for the same level of the MDS stress—a measure of the embedding accuracy—the compressed network has fewer dimensions than the uncompressed network, suggesting that the compressibility is a somewhat general property: The compressibility by a g-network has led to the compressibility by the MDS. Interestingly, the MDS features of the compressed network seem to resemble the Gabor filters (Fig. 2*F*) whereas those from the uncompressed network seem more like filters tuned to individual digits (Fig. 2*G*).

To illustrate how high innate performance could provide an evolutionary advantage, we formulated a simple model in which an organism’s survival to reproductive maturity—its fitness—was proportional to its performance on this task. In this model, the probability p(t) that the organism is still alive at time t is given by

[2] $$\begin{array}{c} p(t+1)=p(t)[1-\alpha (1-c(t))]. \end{array}$$

Here, c(t) is the rate of correct performance at the time t and 0<α<1 is a parameter that determines the contribution of this trait to survival. This model thus relates the correct performance on the task to survival (Fig. 2*H*). Fig. 2*I* shows how, over successive generations, the fraction of individuals with high innate performance increases at the expense of individuals who rely solely on learning to acquire fitness. As expected, over several dozen generations, the individuals with higher innate fitness dominate the population, completely supplanting those initialized tabula rasa, who must learn everything from the environment. Many factors in the real world could serve to complicate this simple model, which for example does not explain the prolonged period of postnatal helplessness of mammals. Nonetheless, the model provides an intuition for why evolution might be expected to maximize high innate performance.

We hypothesized that passing the wiring diagram through the genomic bottleneck would extract the most useful and important features of the connectivity and enable generalization to related tasks. To test this idea, we used the related Fashion-MNIST dataset (below, we will call it F-MNIST for brevity), which has the same format as the MNIST dataset but consists of ten different categories of clothing (shirts, shoes, etc; Fig. 2*J*). Disappointingly, we observed no enhancement of F-MNIST learning upon initializing weights using a MNIST-trained g-network. Indeed, the p-network adapted from the MNIST dataset showed somewhat slower learning than a naive network (Fig. 2*K*), as though the network first had to unlearn the MNIST dataset before learning the F-MNIST. We hypothesized that this failure to generalize across visual recognition tasks was due to overfitting on the specifics of the MNIST dataset, due to the relative simplicity of the tasks and the network used to solve them: Both of these datasets are too simple to require learning general properties of images that can be transferred to novel visual problems.

### Application to the CIFAR-10 Dataset.

**Fig. 3.**
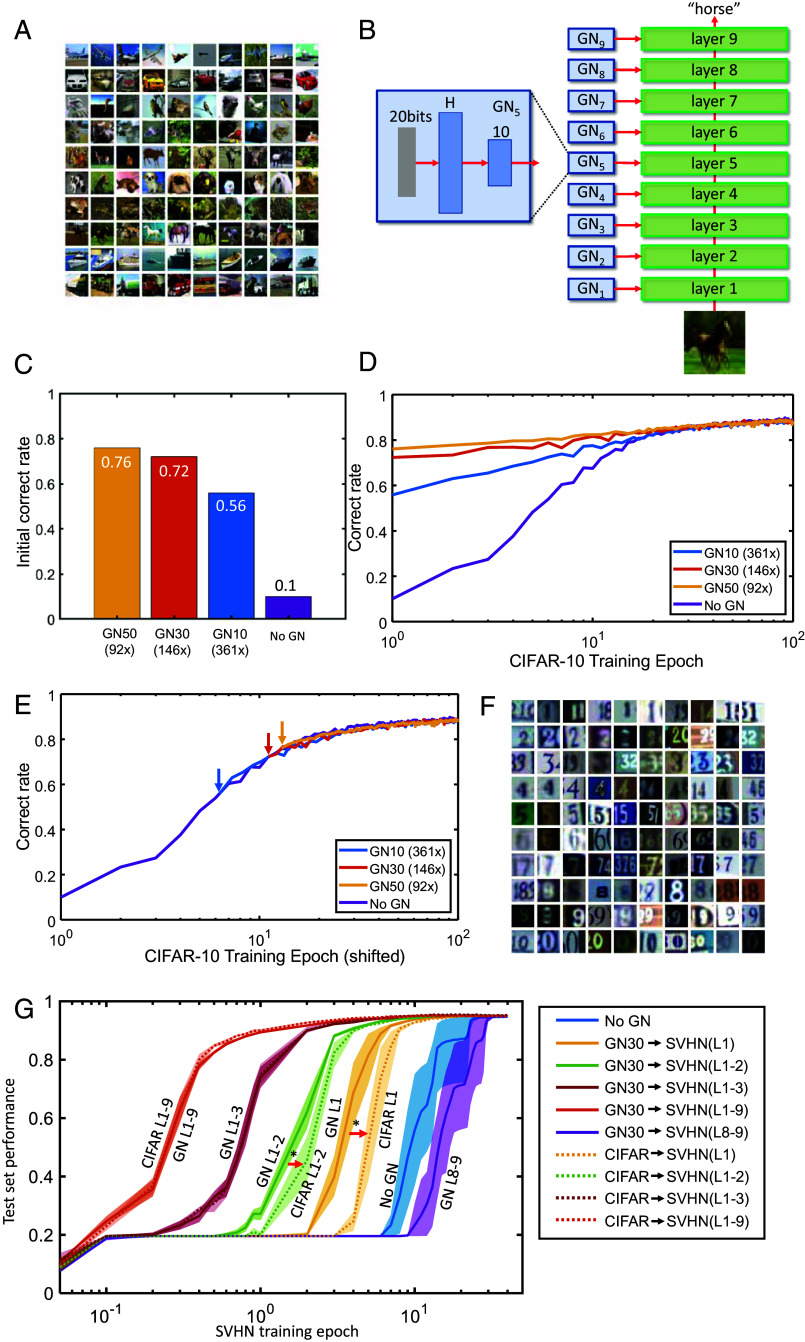
Genomic bottleneck approach to CIFAR-10. (*A*) Examples of images from the CIFAR-10 dataset. (*B*) To classify the CIFAR-10 images, we used a nine-layer convolutional network. Each layer was created by individual g-networks. (*C*) The p-network achieves excellent tabula rasa performance even for greater than 100-fold compression. (*D*) Dynamics of learning for different levels of compression. (*E*) The learning rate of the compressed networks is the same as tabula rasa networks. For each level of compression, the curve in D is shifted to the tabula rasa curve. (*F*) Example of images from the SVHN dataset. (*G*) Transfer learning to SVHN dataset. Results are shown as training curves on the SVHN dataset for networks initialized using different sets of layers transferred from the CIFAR-10 dataset as indicated. For example, the green solid curve (GN L1-2) shows results for layers 1 and 2 initialized using g-networks trained on the CIFAR-10 data, while the remaining layers are initialized randomly (shaded regions show SD of the mean). For the CIFAR-10 L1-2 curve, layers 1 and 2 were initialized by direct transfer from the CIFAR-10 dataset. GN L1-2 is shifted compared to CIFAR L1-2 (red arrow) indicating the advantage of our approach. A similar feature is observed for layer one transfer curves (orange). Stars indicate statistical significance (ANOVA, *P* = 0.015 for L1 and *P* = 0.0005 for L1-2). The GN L8-9 curve shows worse performance than naive training (No GN), similar to Fig. 2*K*.

To test whether there are conditions where the genomic bottleneck might extract features that generalize over multiple datasets, we applied the algorithm to a more complex problem that requires a deeper network. We used the CIFAR-10 dataset, which consists of 60 k color images drawn from 10 categories such as airplanes, cars, and cats (Fig. 3*A*). For the p-network we used a standard 9-layer convolutional neural network (CNN) architecture with about $1.4\times 10^{6}$ weights (*Methods*). We compressed each CNN layer of the p-network with a separate g-network (Fig. 3*B*). Similarly to MNIST, the compressed CIFAR-10 network reached high performance. GN50, a network with 92-fold compression, achieved initial performance of 76% (vs. naive 10%), fairly close to the fully trained performance of 89% (Fig. 3 *C* and *D*). Excellent compression was also achieved with two larger datasets, ImageNet-1K and Caltech-256, confirming the scalability of our approach (*SI Appendix*, A). Thus, as with MNIST, the g-network achieved approximately two orders of magnitude compression while maintaining good initial performance.

The learning dynamics (Fig. 3*D*) demonstrate the utility of genomic compression for achieving enhanced initial performance. However, it was not clear whether this was also associated with faster learning. To our surprise, genomic compression did not affect the learning trajectory; the only speedup was due to the higher initial performance, as though the p-network was “hot-started” by the g-network (Fig. 3*E*). Thus genomic compression, at least under these conditions, did not affect the learning rate.

To assess whether the structure that g-networks extracted from the CIFAR-10 dataset could be useful for other datasets, we tested transfer from the CIFAR-10-trained network to a related problem. We used the Street View House Numbers (SVHN) dataset, which contains images of house numbers in a format similar to the CIFAR-10 dataset (Fig. 3*F*).

We first confirmed the effectiveness of a standard algorithm for transfer learning. We trained the p-network on the CIFAR-10 dataset without compression and then used the p-network’s weights as a starting point for the SVHN training (CIFAR-10 transfer). As expected, this procedure accelerated learning, reducing training time from about 20 training epochs (Fig. 3*G*, blue line, “No GN”) to a single epoch (Fig. 3*G*, “CIFAR L1-9”). This implies that the CIFAR-10 dataset contains features similar to the SVHN dataset.

We next compared the standard transfer learning algorithm to an algorithm based on the genomic bottleneck. To achieve transfer with the genomic bottleneck, we used g-networks to generate a p-network as described above and then used this p-network as an initial condition for SVHN training (g-network mediated transfer). Remarkably, the performance of g-network mediated transfer was indistinguishable from the standard approach (Fig. 3*G*, orange solid line, “L1-9”), even though in this case the number of transferred parameters was 92 times fewer. These results indicate that whatever is crucial for transfer from the CIFAR-10 to SVHN is captured by the nearly 92-fold smaller g-network.

To further dissect the consequences of genomic compression, we examined the effect of transferring one or a few layers at a time. When only the first two lower layers (layers 1 to 2) were transferred from the CIFAR-10 to SVHN, while randomly initializing the remaining layers, genomic transfer yielded faster learning than direct transfer (Fig. 3*G*, red arrows). For example, when layer 1 was initialized with g-network, 50% performance was reached in about 3.2 training epochs, while similar levels were only achieved in 4.8 epochs using direct CIFAR-10 transfer—a 1.5-fold difference. Thus, it appears that the lower layers of the network contain features that generalize across datasets, and these features are extracted well using our compression algorithm. On the other hand, transferring the last two layers of the network resulted in slower training compared to the naive case, a result reminiscent of our MNIST-to-F-MNIST transfer (Fig. 2*K*). This result implies that the last two layers of the CIFAR-10-trained network contained features that are specific to the dataset and were not useful for the recognition of the house numbers in the SVHN data.

Taken together, these findings demonstrate that g-networks can extract structure that is generalizable across datasets. Compression with g-networks yields performance that is comparable to—and in some cases better—than simple uncompressed weight transfer, indicating that g-networks identify a special subclass of p-networks that are compressible and capture essential structure of the data (Fig. 1*D*). This enhancement is particularly evident in the rate of transfer of the lower layers in deep nets (Fig. 3*G*). Interestingly, the receptive fields of neurons in the lower visual system show substantial similarities between different species, while higher layers are more specialized (25). This parallel with our results suggests that the early visual system may have extracted a simple yet potent set of features while subject to genomic bottleneck-like constraints.

## Reinforcement Learning

The results described so far demonstrate the efficacy of genomic compression in the context of supervised learning. However, supervised learning is unlikely to play a major role in animal behavior (2). We therefore turned our attention to reinforcement learning paradigms, in which an agent seeks to maximize its reward in a given environment by taking actions based on its current state and its history of actions and rewards. The actions are determined by a policy that maps the agent’s state to actions. Learning in this context consists of adapting the policy. Many of the most successful modern approaches use ANNs to implement the policies (26).

**Fig. 4.**
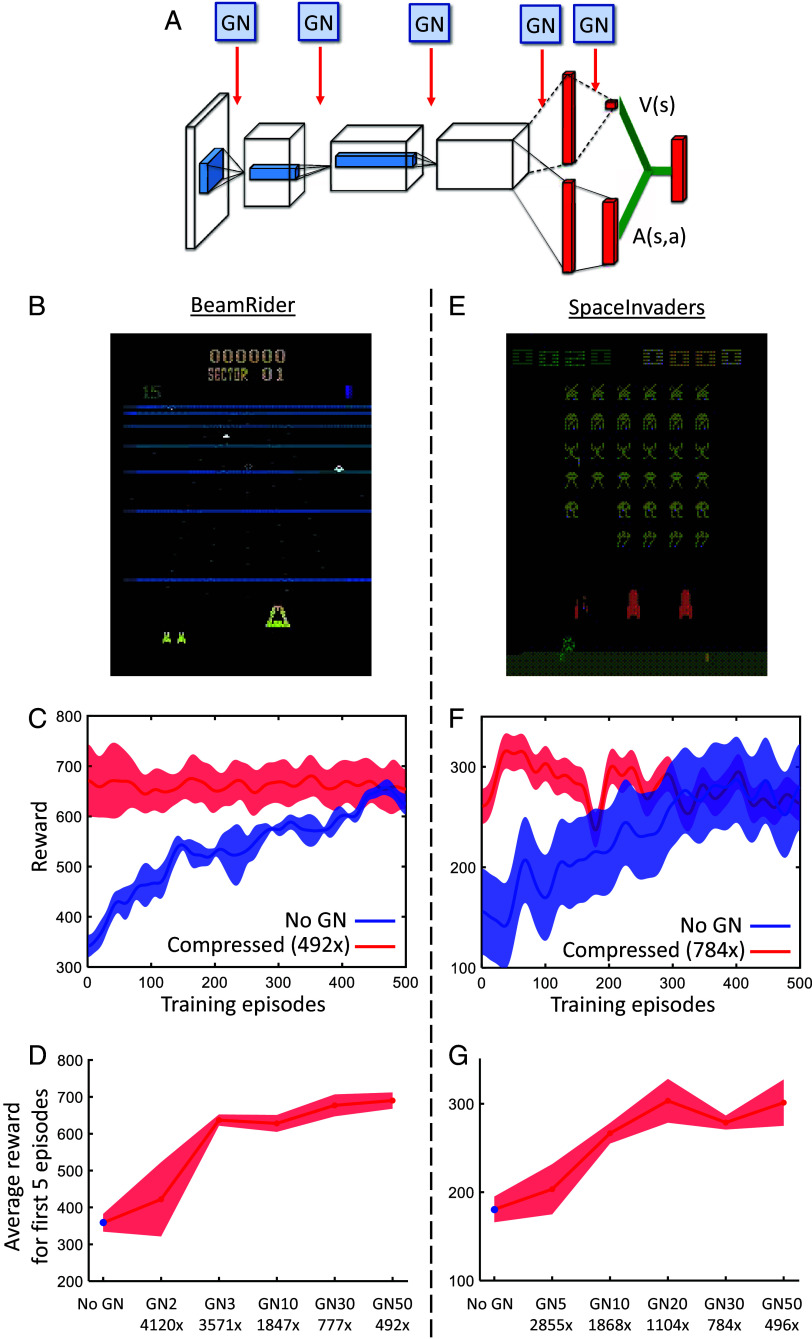
Genomic bottleneck approach to reinforcement learning. (*A*) The network architecture of p-network consists of 5 layers, each of which was compressed by a corresponding g-network. (*B*) Application to the video game BeamRider, in which the goal is to shoot down enemy ships. (*C*) Dynamics of learning for an uncompressed network (*blue*) is slower than for a 492-fold compressed network (*red*), which achieves nearly perfect performance on episode 0. Shaded regions show the SD of the data. (*D*) Performance over the first 5 episodes as a function of compression. Nearly perfect initial performance is achieved by the 3570-fold compression. (*E*-*G*) Analogous modeling for the video game SpaceInvaders.

We first tested the genomic compression algorithm on the ANN-based policies used for solving BeamRider (27), a video game (Fig. 4*B*). In this task, the input is a set of 80×80 pixels and the output is one of 9 actions (e.g. move left, fire, etc). We used the Dueling Deep Q-Network (28) to train a standard p-network with $3.3\times 10^{6}$ parameters (Fig. 4*A*). With training, the performance doubled after several hundred episodes (Fig. 4*C*). We then compressed the p-network 492-fold using a g-network with about $10^{4}$ parameters. The initial performance of the compressed network indicated that the g-network was able to capture almost all of the structure inherent in the connections of the p-network. We then tested greater levels of compression and found that the innate performance of the compressed network remained excellent up to about 3,500-fold compression (Fig. 4*D*). Similar results were obtained for another video game, SpaceInvaders (Fig. 4*E*–*G*). These results show that the genomic compression approach is not limited to supervised learning, but can be readily extended to a reinforcement learning setting.

**Fig. 5.**
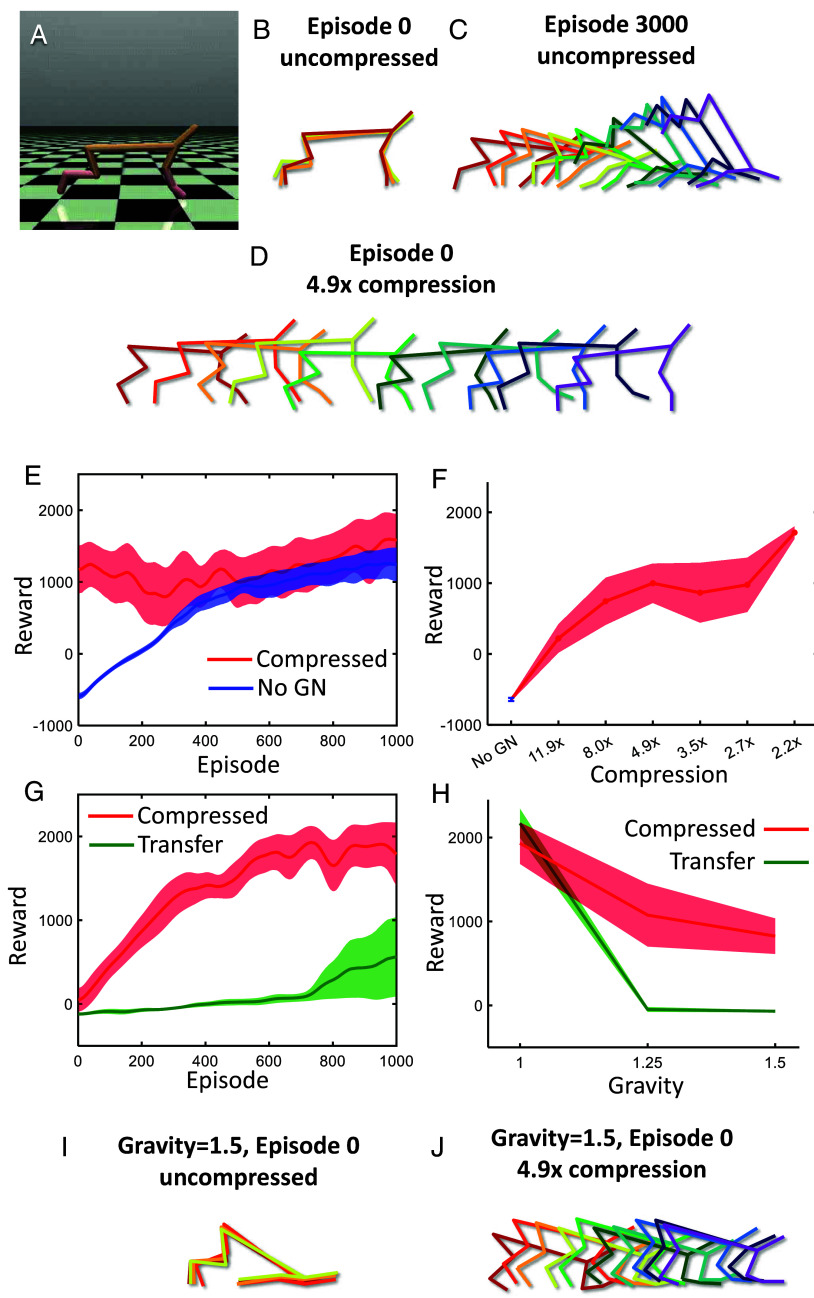
Genomic bottleneck applied to HalfCheetah. (*A*–*D*) Snapshots of sample episodes. (*A*) Task screenshot. (*B*) Upon initialization without compression, the HalfCheetah typically flails about, largely in place. (*C*) After 3,000 episodes of training, the agent finds an effective but often unconventional strategy for moving forward. In this example, the strategy involves intermittent sliding on its chin. (*D*) The agent trained with the genomic bottleneck approach adopts an effective and conventional strategy even in episode 0. (*E*) Learning time course for 4.9-fold compressed (*red*) vs. randomly initialized (*blue*) networks. The shaded region shows the SD of the data for four independent training runs. (*F*) Initial performance of the HalfCheetah network averaged over the first 5 episodes as a function of compression. Performance is shown for individual genomic networks (dots) and the average (solid line). (*G*) The results of weight transfer to the environment with higher gravity. Learning time course for 4.9-fold compressed (*red*) vs. uncompressed (one fold, direct transfer) (*green*) networks. (*H*) Performance averaged over the first 400 training episodes as a function of the gravity scale. Dots/lines show results for individual networks and their average. Colors are the same as in (*G*). (*I* and *J*) Skeleton diagrams showing subsequent time steps for networks initialized by direct transfer (*I*) and 4.9-fold compressed g-network (*J*) in the increased gravity environment.

We next tested the genomic compression algorithm on a more challenging reinforcement learning task, HalfCheetah (Fig. 5*A*). In this task, a simulated cheetah must learn to maximize its forward velocity in a simulated physics environment, MuJoCo (29). Here, the state space consists of the positions, angles, and velocities of 8 joints, and the continuous action space consists of the forces applied to those joints. With a random initialization, the cheetah cannot move forward (Fig. 5*B*). After several thousand episodes of training, the cheetah sometimes learns to move forward conventionally, but typically adopts unconventional solutions such as tumbling on its head or flipping upside down and gliding along its back (Fig. 5*C*), a phenomenon sometimes referred to as “reward hacking.” Such solutions often yield rewards comparable to those obtained by conventional solutions and can be viewed as overfitting.

The genomic bottleneck algorithm was able to achieve excellent compression on this task. After 4.9-fold compression, performance on episode 0 is excellent, approaching asymptotic performance (Fig. 5*E*). Initial performance on episode 0 declined only modestly with increasing compression (Fig. 5*F*). Thus, the genomic compression algorithm could be effectively applied even in the more challenging setting of the HalfCheetah.

Interestingly, solutions obtained from p-networks initialized by g-networks did not appear to engage in reward hacking, unlike those obtained following random initialization; following compression, only conventional strategies without tumbling and flipping were learned (Fig. 5*D*). This suggested that compression was acting as a regularizer, discouraging overfitting by reward hacking. To further explore this, we tested performance in a modified environment in which gravity has been increased by 50%. (In the MuJoCo simulation environment, gravity can be conveniently modified with a single parameter, which can be viewed as a surrogate for changes in body size that would occur over an animal’s lifetime). In this modified environment, initial performance at episode 0 even for the p-network initialized by a g-network is poor, but performance improved relatively quickly after several hundred trials (Fig. 5*G*, *red* and *J*). By contrast, conventional transfer learning, in which the fully trained uncompressed network is used in the new environment, learned much more slowly (Fig. 5*G*, *green*; *H* and *I*). The superior transfer learning observed with the genomic bottleneck approach arises because the reward-hacked strategies observed in networks trained without compression do not generalize well to the new environment, so the agent must first unlearn these unconventional strategies. Taken together, our results suggest that the genomic bottleneck approach can be effectively applied to both supervised and reinforcement learning problems.

## Discussion

We have proposed that a genomic bottleneck arises inevitably because of the need for a relatively low-capacity genome to specify the complex neural circuits required for innate behaviors. We argue that under a wide range of conditions, there is evolutionary pressure for organisms to be born with as much innate ability as possible, and thereby to maximize their fitness at birth (Fig. 2*H*). This leads to a model in which genomes, and the circuits they encode, are co-optimized in nested loops: an inner loop corresponding to “learning” in animals, and an outer loop corresponding to “evolution.” Our results suggest that dividing the usual machine learning problem into such nested loops linked by a low-information bottleneck serves as a regularizer on the resulting neural circuit, guiding it to find simple circuit motifs that can be reused and can adapt to changes in the environment.

The genomic bottleneck can be viewed as a constraint forcing lossy compression of the weight matrix (Eq. **1**). The idea of minimizing the description length, or Kolmogorov complexity, of the weights has a long history (30, 31). Although the genomic bottleneck algorithm was motivated by the considerations of the relative size of the genome and the connectome, it has close parallels with the “information bottleneck” method (15). We hypothesize that, by squeezing the neural circuit diagram through a much smaller genome, evolution has extracted the most useful and important network motifs.

To perform tasks, the compressed (genomic) representation must be uncompressed into a functional network through a process analogous to neural development. For the reasons of efficiency, we have used gradients to optimize both the inner and outer loops. Evolution, which can be viewed as a form of optimization that does not exploit a gradient, is in general a relatively slow and inefficient algorithm, successful because it operates on massive numbers of individuals in parallel over hundreds of millions of years. The feedback in our algorithm that guides the gradient from each generation—the fact that the trained weight matrix in the kth generation is used to modify the genome in the (k+1)st generation—can be viewed as a form of Lamarckian evolution, and is, as such, biologically unrealistic. The net effect of our approach, however, is similar to Darwinian evolution. Our algorithm can also be seen as an implementation of the Baldwin effect (32, 33), according to which, if the ability to learn a particular behavior rapidly confers a selective advantage, that ability would, throughout evolution, be “genetically assimilated” and might appear to have arisen through a Lamarckian process.

We developed a genomic bottleneck algorithm that could achieve several orders of magnitude compression on standard supervised and reinforcement learning benchmarks. Although it might seem surprising that these networks could be so highly compressed with relatively little loss of performance, our results are consistent with considerable literature on network compression (34). For example, a standard technique—weight pruning—can eliminate 90% of parameters with minimal loss of accuracy (22, 23). Network pruning can be viewed as a method of network compression, complementary to the genomic bottleneck mechanism considered here. Convolutional neural networks (18) can be viewed as an example of network pruning prior, where each neuron only connects to a small fraction of other neurons in lower layers. Another example is the lottery ticket hypothesis, according to which the number of weights in a well-performing network can be substantially reduced by discovering “winning ticket” sparse subnetworks (35). Cortical networks are inherently sparse, with each neuron connecting to only a minute fraction of other cortical cells. Evolution may have selected sparse and functionally important connections due to physical constraints, such as space and time limitations (36).

Even after sparsification, the organization of cortical connections cannot be encoded in the genome with a single-neuron precision. For example, sparse connectivity, such as that produced by the lottery ticket, will require $H\;N_{s}\log N_{s}$ bits to encode ($N_{s}$ is the number of synapses), which amounts to about 260 TB in case of cortical connectivity(9). Thus, pruned connectivity does not by itself resolve the discrepancy between cortical and genomic information capacities. This implies that other mechanisms must be used to further compress pruned networks. Similarly, the approach based on HyperNetworks (21) trains lower-rank representations of the weight matrices. Such an algorithm should be able to compress a square N×N weight matrix using only HND bits of information, where N and D are the number of neurons in the network and the reduced rank respectively. In its present form, this algorithm is thus limited to compressing a square weight matrix into the amount of space between HN and $H\;N^{2}$ (in the case of DN). Our algorithm offers a fundamentally different solution. In our approach, the vectors representing individual neurons are not expected to be stored in the genome. The minimum size needed to store a p-network in the genome scales with the number of parameters in the g-network. The latter, in the minimal case, is determined by the size of g-net inputs, i.e. the size of binary vectors representing individual neurons in the p-network. A p-network with N neurons requires $\log_{2}N$ binary tags to specify each neuron uniquely, so the input to the g-network is $\log_{2}N$ units (binary digits) for each neuron. Thus, the minimal size of the g-network needed to encode the p-network of size N scales as HlogN. Thus, in our approach, the small amount of information parameterizing the g-network can be used to encode even very large p-networks making it distinct from other approaches.

Our results contribute to a growing literature highlighting the importance of inductive biases in machine learning. Much of this literature is focused on achieving faster learning. Perhaps the most successful examples are convolutional neural networks (18), which exploit the translational invariance of images with an architecture inspired by the structure of receptive fields in the early sensory cortex (37). However, the present work—inspired by evolutionary constraints (Fig. 2*D*)—is focused not on faster learning but rather on enhanced initial performance, a goal that has received comparatively less attention (but see ref. 38). Indeed, in our experiments, we find that genome-initialized networks start at a higher level of performance but then follow the same trajectory as randomly initialized networks (Fig. 3*D*). Although these results highlight the potential dissociation of two distinct processes—better initial performance and faster learning through inductive biases—there is likely strong evolutionary pressure to maximize both.

The relative importance of genomically encoded innate structures in determining specific human abilities such as language has been hotly debated, but the importance of innate factors to the behavior of other animals is less controversial. For both humans and animals, the better question is usually not whether a behavior is innate, but rather how innate and learned factors interact. For example, the propensity to form “place fields” in the hippocampus is innate—a map of space emerges when young rat pups explore an open environment outside the nest for the very first time (39)—but the content of place fields is learned, as new place fields form whenever the animal enters a new environment. In this example, it appears that, as suggested by our experiments, innate performance has been maximized by providing a scaffolding for place fields to appear.

The genomic bottleneck takes its inspiration from fundamental considerations about the evolution and development of brain circuits. Although the genomic bottleneck algorithm builds on existing machine learning techniques and yields surprisingly effective performance, we have not attempted to optimize this approach to compete with state-of-the-art benchmarks. The bottleneck framework is potentially quite rich and could be extended in several directions. For example, we have not explored variations in the structure of the genomic network, e.g. by imposing a sparseness constraint. Such a constraint would have the physical interpretation of limiting interactions among surface neuronal markers. Similarly, at present, each layer is compressed with its genome, but it would be natural to attempt to extract regularities among layers by encoding them with a single genome. Furthermore, in the current formulation, the decoding of the genomic network is deterministic, whereas developmental rules are often stochastic, so the decoding framework might be generalized to include rules like “let each neuron connect on average to 10% of nearby neighbors” or a more complex stochastic rule (40). Finally, the framework could be extended to co-optimize the learning rules and the wiring.

## Methods

### MNIST/F-MNIST Dataset.

For the MNIST and F-MNIST datasets, we used a fully connected 2-layer network that included 800 hidden layer ReLU units (41). We did not use data augmentation for simplicity and our network could be trained to 98% performance on testing data. The number of parameters in the MNIST network was, therefore, $28^{2}\times 800+800\times 10$ weights and 800+10 biases amounting to the total of 636,010. We used three g-networks to encode two weight matrices and one bias vector for the hidden layer. The ten biases for the output layer were not compressed since the corresponding g-network would include more than ten parameters. The structures for various configurations of the three g-networks are listed in Table 1. The schematic of the structure of the g-network for the MNIST dataset is shown in *SI Appendix*, Fig. S.1*B*.

**Table 1.** The structure of g-network for the MNIST dataset

g-network name	g-network structure	Bias net structure	g-network parameters	MNIST parameters	Compression
GN5	(20−10−5−1)×2	10−5−1	613	636,010	1,038×
GN20	(20−20−10−1)×2		1,353		470×
GN30	(20−30−10−1)×2		1,973		322×

Each neuron was described by a binary label of length 10. For the neurons in the input image, the label encoded two coordinates of the neuron’s position in the image, 5 bits for the x and y coordinates. Both coordinates were represented by the Gray code. The neurons in the hidden layer were represented by simple binary codes ranging from 1 to 800 since their order is of no particular importance. The ten neurons in the output layer were encoded by the one-hot vector of 10 bits. Each neuron in the networks was therefore described by a ten-bit label. The inputs into each of the two g-networks that generated weight matrices represented pairs of neurons and had a length of 20 bits. The output of g-network is the value of the corresponding weight between two input neurons and was a single real number (Table 1). For the network generating biases for the hidden layer, the input contained the binary label for the neuron and was therefore 10 bits long.

To train g-networks, we developed the intermittent training paradigm. Before the first iteration, the p-networks are initialized randomly. In each iteration of this method, we train the p-network using a subset of images. For the MNIST network, this training used 10,000 images from the training set or 1/6th of the epoch. This yielded a higher-performance p-network with the set of weights described by matrix $W_{n}$. We then train g-networks to approximate this weight matrix by backpropagating the difference between the g-network output (${\tilde{W}}_{n}$) and $W_{n}$. We used different numbers of weights to train each of the three g-networks on each iteration ($10^{5}$, $10^{4}$, and $10^{4}$ for hidden layer, output layer, and hidden layer biases g-networks, respectively). This amounted to about 1/6th of all weights and further accelerated training in each generation. We then used the adjusted g-networks to generate the complete set of p-network weights ${\tilde{W}}_{n+1}$ that served as initial conditions for the next generation (*SI Appendix*, Fig. S.2*A*). This set of iterations mimicked real biological evolution as it alternated the generation of p-networks from g-networks, analogous to neural development, and improvement of p-networks similar to natural selection. We repeated these iterations 500 times to achieve the asymptotic performance. We provide the details in *Algorithm 1*.

### CIFAR-10/SVHN Datasets.

In this example, we used an all-convolutional 9-layer implementation of network (42) (*SI Appendix*, Fig. S.1*C*). Between layers 3 and 4, we included the dropout layer with 50% dropout probability. The network could be trained to 89% correct rate without data augmentation. Each layer in this 9-layer CNN was generated via two g-networks, one for the weight matrix and one for the biases. To provide inputs into the g-networks for weights, we described positions in the weight matrix by a 20-bit binary number. For the lowest 8 layers, the binary number was composed of 2 + 2 bits containing Gray code representation for the input coordinates within the CNN kernel, 8 bits representing the input filter type, and 8 bits representing the output filter type. The latter two components were formed as consecutive binary numbers since input/output filter identity is not expected to form a continuous topographic space. In this representation, the input neurons were identified by a 12-bit binary label (2 + 2 Gray + 8 binary) while the output neurons are identified by the 8-bit label. The binary labels for the last CNN layer were composed of 1 + 1 binary Gray (dummy) bits representing two coordinates within the kernel, an 8-bit binary number representing the input filter, and a 10-bit one-hot vector encoding the output class. The bias networks for the first eight layers received an 8-bit label encoding the output filter. The bias network for the last layer received the one-hot vector encoding one of the ten output classes. The structures of different g-networks used are summarized in Table 2.

**Table 2.** The structure of g-network for the CIFAR-10 dataset

g-network name	g-network structure	Bias net structure	g-network parameters	CIFAR-10 parameters	Compression
GN10	(20−10−10−1)×9	8−10−1	3,798	1,369,738	361×
GN30	(20−30−10−1)×9		9,378		146×
GN50	(20−50−10−1)×9		14,958		92×

To train the 18 g-networks described above, we used the intermittent training strategy described for the MNIST network. We used minibatch sizes of 10, 100, and 1,000 for bias g-networks for layers 1 to 3, 9, bias g-networks 4 to 8, and all weight matrix g-networks, respectively. We used the minibatch size of 128 to train the CIFAR-10 network. We used the stochastic gradient descent (SGD) optimizer for the CIFAR-10 network with a learning rate of 0.05 and a momentum of 0.9 for stability. We used the adaptive momentum (ADAM) optimizer for all g-networks. In each iteration, we first trained the CIFAR-10 network using 10 complete epochs, i.e. 10 complete sweeps through the entire training set of images. In the second step, we trained weight and bias g-networks using 2 and 10 epochs respectively (we trained g-network weight networks for layers 1 and 9 using 12 epochs in each iteration) to match the CIFAR-10 network adjusted weights resulting from the first step. This sequence of two steps was repeated 500 times.

Because our network was relatively deep (9 layers), we encountered a problem with the initialization of g-networks. Indeed, if g-networks are initialized randomly, they produce p-networks that are far from the optimal fixed point. We found that such p-networks are impossible to train. This problem is exacerbated in moderately deep p-networks due to the exponential divergence of initialization errors from layer to layer. In practice, such p-networks return zero activations, which yield no gradients of weights. To solve this problem, we implemented the weight annealing strategy. In each iteration of our algorithm (out of 500), before the p-network was trained, the weight matrices and biases of the p-network were combined from the results of the CIFAR-10 training in the previous iteration, $W_{n-1}$, and the weights generated by g-network in the previous iteration, ${\tilde{W}}_{n-1}\left(G\right)$:

[3] $$\begin{array}{c} W_{n}=\epsilon \left(n\right)W_{n-1}+{\tilde{W}}_{n-1}\left(G\right)\left[1-\epsilon \left(n\right)\right] \end{array}$$

The coefficient ϵ(n) determined the degree to which the inputs from g-networks affect the p-network’s weight matrix. If ϵ(n)=1, the weight matrix of the p-network is entirely determined by the result of the previous iteration of the CIFAR-10 training and is not sensitive to the inputs from the g-network. In the other extreme, when ϵ(n)=0, the values of p-network weights and biases are entirely generated by the g-networks. We, therefore, assumed that ϵ(n)≈1 at the beginning of training, when g-networks are naive, and ϵ(n)→0 at the end of training. We adopted an exponential annealing schedule with ϵ(n)= exp(−n/λ), where parameter λ=20 determined the number of iterations in the intermittent training over which the g-networks are assumed to be naive and irrelevant, and initialization using g-networks is assumed to be detrimental. Since, in our approach, the total number of iterations is 500, the initialization period is negligibly short compared to the whole training (λ≪500).

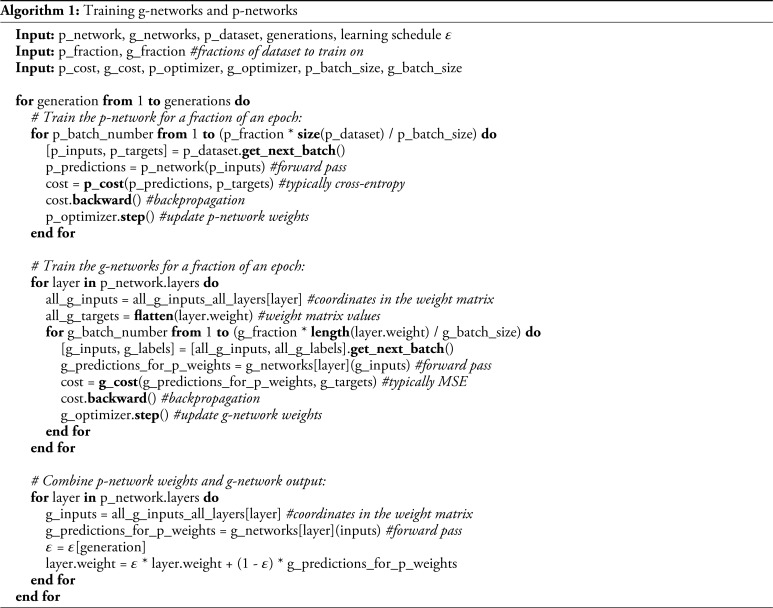

### RL Methods.

We performed experiments on two reinforcement learning tasks from the OpenAI Gym environment (43): BeamRider and HalfCheetah. The details of the experiments follow.

For the first experiment, we tested the genomic bottleneck on solving the BeamRider task, which is part of the ATARI benchmark (27). The task in the BeamRider is to traverse a series of sectors where each sector contains 15 enemies and a boss at the end. Additionally, the agent needs to avoid or destroy the debris coming its way. The agent is equipped with three torpedoes that can be used to kill the enemies or destroy the debris.

We used the pixel images of ATARI frames as our state space. ATARI frames are 210×160-pixel images with a 128 color palette. This makes it computationally expensive to use it directly as input to the network. To reduce the dimension of the input, we performed the following preprocessing steps on the images: conversion of the RGB image to grayscale, cropping it to get an image of size 190×144, and finally resizing it to the size 84×84. The action space is of size 9 with various possible actions: fire up, fire, up, left, right, left fire, right fire, up left, up right, and do nothing. The score obtained while playing the game was used as the reward signal.

For the p-network, we used Dueling Deep Q Network (Dueling DQN) (28) to train them on this task. Dueling DQN breaks the calculation of the Q-value into two parts. Q(s,a)=V(s)+A(s,a), where V(s) represents the value of state s and A(s,a) represents the advantage of performing an action a while in the state s. The value of a state is independent of action. This aids us in avoiding the overshoot of the Q-value that otherwise occurs in deep Q networks (44). Since the states are independent of action, action would not have a high Q-value to train on and thus Q-value would not overshoot. This makes the training smooth and less time-intensive.

The p-network architecture includes three 2D convolutional layers and three fully connected layers with a total size of ∼3 M parameters. The input to the Dueling DQN is our preprocessed image of size 84×84 and the output is the Q-value associated with each action.

For the second task, we moved to a more challenging task i.e. HalfCheetah (29). One challenging aspect of this task is that the HalfCheetah uses continuous action spaces rather than discrete unlike most of the ATARI games. This leads to a performance deficit in value approximation-based methods like Dueling DQN.

The state-space of the HalfCheetah is of size 17 which contains the position, angles, and velocities of 8 joints (6 hinge + 2 slider joints). The action space is a 6-dimensional continuous space consisting of the torques applied to each hinge joint respectively. Since we want to maximize the velocity with minimum force generation, the reward function needs a combination of two components. The first component provides a reward for velocity and the second component gives a penalty for using more force.

For training the p-network for this task, we need a training method that can deal with continuous action spaces. The class of methods that addresses this domain includes policy gradient methods because they can directly approximate the policy of the agent from the state. Proximal policy optimization (PPO) (45) is one of the most popular policy gradient methods and is heavily used for such continuous action space tasks. One of the main reasons behind this is that policy gradient methods have a convergence problem which is usually addressed by the natural policy gradient. However, in practice, natural policy gradient involves a second-order derivative matrix, making it not scalable for large-scale problems. PPO uses a slightly different approach. Instead of imposing a hard constraint, it formalizes the constraint as a penalty in the objective function. By not avoiding the constraint at all costs, PPO was able to use a first-order optimizer like the gradient descent method to optimize the objective resulting in faster convergence. The p-network architecture of the HalfCheetah consists of three fully connected layers with 6,092 trainable parameters. The input to our p-network is the state vector of the environment and the output is the action the agent should take given that state.

## Supplementary Material

Appendix 01 (PDF)

## Data Availability

There are no data underlying this work.
