# Supplementary material for: Encoding innate ability through a genomic bottleneck
Source: Proc Natl Acad Sci U S A. 2024 Sep 12;121(38):e2409160121. doi: 10.1073/pnas.2409160121 (PMC11420173; doi:10.1073/pnas.2409160121)
Supplement: Supplementary file 1 — Appendix 01 (PDF) [file pnas.2409160121.sapp.pdf]

1 Appendix: Larger models and datasets

In this work, we introduced the genomic bottleneck framework and evaluated some of its properties using the MNIST, Fashion MNIST, and CIFAR-10 datasets. While adequate for the stated purposes, these datasets are not considered challenging. Besides, these datasets provide explicit opportunities for compression (e.g. large areas of non-stimulated pixels in the MNIST and Fashion MNIST and label-specific colors in the CIFAR-10). To evaluate the scalability of the genomic bottleneck framework and its properties, we performed additional experiments on larger models of the ResNet family [App1] trained on larger datasets: Caltech-256 [App2] and ImageNet-1K [App3]. We discuss the related design choices and present the results below.

Neural network models are sensitive to weight initialization – a consideration especially important for deep models such as ResNets. Principled weight initialization schemes prevent the uncontrolled growth/decay of neuronal activities in deep layers and of gradients in early layers of models [App4, App5]. While additional techniques (e.g. batch normalization) help to further stabilize the learning, principled weight initialization is heavily relied upon in the training of neural network models and needs to be accounted for in the genomic bottleneck framework.

Conventional weight initialization techniques specify the variance for initial weights in each layer [App4, App5]. While the uniform distribution is typically used for weight initialization, the shape of the distribution does not play a role in derivations of the optimal variance, likely rendering particular distribution choices to be a historical artifact. To match the conventional variance of the p-network weights at initialization, we follow the standard logic by normalizing the g-network inputs (making them zero-mean and unit-variance) and initializing the g-network weights using the Kaiming rule [App5] with the variance additionally divided by 2 for the first layer. In this rule, we only consider the number of input neurons (as they affect neuronal activations during forward propagations) and not the number of output neurons (affecting backpropagation); thus, we maintain the unit variance of neuronal activations at each g-network layer. We then multiply the output of the last layer by the conventional variance used to initialize the respective p-network layer. As a result, per the central limit theorem, we generate the p-network weights that follow the Gaussian distribution with the conventional variance. Though we could convert these weights to the uniform distribution (e.g. by passing them through the cumulative density function, represented here by the error function), the logic above and our pilot experiments (not presented) render this step unnecessary.

Similarly to forward propagation relying on the magnitude of neuronal activations, backpropagation relies on the magnitude of gradients that shouldn't vanish or explode making the learning slow or unstable [App4]. While the magnitude of gradients is taken care of automatically in p-networks (by using the standard weight initialization, which we reproduced in the previous paragraph), and the gradients in g-networks do not play a significant role in learning (as g-networks are not deep by design), the interface between p-network and g-network backpropagation needs to be considered separately in end-to-end learning (Fig. S.5A). This is because activations of g-networks serve as weights in p-networks, and different normalization rules apply conventionally to activations and weights. Specifically, for convolutional layers, constituting the majority of the ResNet architecture, the expected variance of the p-network gradient equals the (squared) output size of the convolutional layer, while the unit variance is expected in g-networks. To account for this discrepancy, we scale the gradients by the (linear) size of the layer's output before passing them to g-networks. For fully connected layers, no adjustment is needed.

To test the scalability of our approach, we applied the genomic bottleneck framework with the considerations listed above to the ResNet [App1], a backbone of modern computer vision models. For illustration purposes, we used the ResNet-18, the smallest model of the ResNet class consisting of 18 layers with 11.7M trainable parameters. This model contains convolutional layers, fully connected layers (including skip-connections), and batch normalization layers. For training, we followed a simplified protocol that included data augmentation, similar to [App6]. We did not use finetuned learning rate schedules, opting for the ADAM optimizer with the standard learning rate instead. We also did not use cosine annealing as, per our pilot experiments, it did not have a major effect on learning. In g-networks, we did not optimize the compression levels for individual p-network layers. To evaluate different levels of compression, we used the fact that the last (fully connected) layer in ResNet-18 is one of the largest layers in the model, representing a large share of the model's parameters; thus we only changed the compression of this layer.

We conducted the first set of experiments with the Caltech-256 dataset [App2], featuring 30K images belonging to 257 classes (256 image classes plus a separate "clutter" class; Fig. S.5B). When trained directly (p-network only), the model reached 91% classification accuracy on the held-out testing set (Fig. S.5C; compare to the SOTA of 97% [App7]). When trained within the genomic bottleneck framework, the p-network reached 90% accuracy at 23-fold compression and 90% accuracy at 28-fold compression (Fig. S.5C,D), closely matching the uncompressed result on this benchmark. We further tested our framework on the ImageNet-1K dataset [App3], featuring 1M images belonging to 1000 categories (Fig. S.5E). Image classification in this dataset is difficult due to the similarities between some categories (e.g. various dog breeds split into separate classes) and the large size (150+G). When trained directly (p-network only), the model

reached 65% classification accuracy on the held-out validation set (Fig. S.5F; compare to 69% with this model in the original work [App1]). When trained within the genomic bottleneck framework, the p-network reached 57% accuracy at 13-fold compression and 51% accuracy at 24-fold compression (Fig. S.5F,G).

To gauge the achieved compression, we compared the parameter counts and classification accuracies above to respective numbers in other approaches. First, the number of parameters in the ResNet-18 (p-network) is already optimized compared to preceding models. The reduction in parameter counts was achieved by excluding heavy fully connected layers at the end of the model (compare 11.7M parameters in the ResNet-18 [App1] to 62.3M parameters in the AlexNet [App8] and 138M parameters in the VGG-16 [App9]). Second, the existing compression frameworks mostly focus on the compression of pretrained models. Successful approaches, optimizing per-layer compression, are highly efficient within the range of 2-fold compression where their performance decay is as little as a few percent [App10]. The performance of these models, however, drops after 3-fold compression (20+% decay) [App10]. At the same time, the genomic bottleneck framework at 13-fold and 24-fold compression shows 8% and 14% decays in classification accuracy respectively (Fig. S.5F,G) suggesting that it finds an intersection between the spaces of high-performance models and compressible models, not generally achievable by direct training of p-networks whose regularity is not enforced and thus may be limited. Third, the same performance-compression tradeoff may not be achieved by directly training a smaller model (e.g. a model with a reduced number of layers). To test this proposition, we trained a ResNet-6 model. Despite the higher parameter count compared to our compressed models (5-fold vs 13-fold compression), its classification accuracy was lower (50% vs 57% respectively; Fig. S.5G). This observation suggests that the genomic bottleneck framework is capable of learning non-trivial patterns in data that require an increased depth of models but take advantage of their symmetry.

ResNet Methods

To evaluate the genomic bottleneck framework, we used the Caltech-256 (via Torchvision 0.16.1) and ImageNet-1K [App3] datasets. We used the standard ILSVRC2012 training and validation sets in ImageNet-1K; for Caltech-256 we split the data into training and testing sets by randomly sampling 80% and 20% of images respectively. We preprocessed the testing set images by converting grayscale images to RGB via copying wherever it applies, resizing (to 256 x 256 x 3 pixels), cropping (to 224 x 224 x 3 pixels, centered), and normalizing (mean: [0.485, 0.456, 0.406], standard deviation: [0.229, 0.224, 0.225] for red, green, and blue channels respectively). For training set images, the first two steps were replaced with the random resized crop (to 224 x 224 x 3 pixels) and random flip to augment the data.

To classify the images in these datasets, we trained ResNet models using PyTorch 2.1.1 on an NVIDIA Tesla V100S GPU (32 Gb). Unless mentioned otherwise, we used the ResNet-18 model (Torchvision 0.16.1 implementation). In ResNets, we disabled the learning of the batch normalization parameters including the momentum. We estimated the batch mean and variance directly from training or testing data. We also adjusted the size of the last layer to match the number of classes in datasets (1000 for ImageNet-1K and 257 for Caltech-256). Additionally, we implemented a ResNet-6 model where we reduced each basic block to a single convolutional layer (with filter dimensions of 64 x 64, 64 x 128, 128 x 256, and 256 x 512 respectively). For p-network training (with no g-networks), we used the ADAM optimizer with a learning rate of 0.001 in batches of 1024 for 1000 epochs (Caltech-256) or 100 epochs (ImageNet-1K). We randomly sampled the data to form batches during training and did not permute it during testing for reproducibility.

To compress the models, we implemented g-networks to generate ResNet-18 layers as follows. For convolutional and downsample layers (both implemented as convolutional layers), we used fully connected g-networks (hidden layers sizes: 100, 100, and 50 neurons; nonlinearities: ReLU except for the last layer) with one-hot inputs corresponding to the spatial dimensions and binary number inputs corresponding to the filter dimensions of the ResNet convolutional kernels. For the last fully connected layer we either used the same g-network structure as for convolutional layers (but with binary numbers for the input dimension and one-hot codes for the output dimension; total compression: 28x for Caltech-256, 24x for ImageNet-1K) or left it uncompressed (total compression: 23x for Caltech-256, 13x for ImageNet-1K). We also left all other types of parameters, including the biases, uncompressed. We normalized the inputs to g-networks (mean: 0 and variance: 1) and initialized the weights of g-networks using the Kaiming rule (with the variance divided by 2 for the first layer) where we only considered the numbers of inputs (and not outputs) to maintain unit-variance neuronal activations across g-network layers. We multiplied the outputs of the last g-network (linear) layers by the standard deviation of the weights in respective ResNet-18 layers as measured at the initialization (to match the Kaiming initialization for the ResNet-18).

We trained these g-networks and ResNet-18 end-to-end using ADAM optimizers with a learning rate of 0.001. For convolutional and downsample layers, we scaled the gradients between the ResNet and g-networks by the spatial size of respective convolutional kernels to maintain a near-unit variance in the backpropagated signal. For the ResNet-18 we used the batch size of 750 images to fit the GPU; we trained g-networks in batches equal to the sizes of respective ResNet-18 layers. We trained the models for 10000 epochs (Caltech-256) or 2000 epochs (ImageNet-1K).

Appendix references

[App1] Kaiming He, Xiangyu Zhang, Shaoqing Ren, and Jian Sun. Deep residual learning for image recognition. In *Proceedings of the IEEE conference on computer vision and pattern recognition*, pages 770–778, 2016.

[App2] Gregory Griffin, Alex Holub, and Pietro Perona. Caltech-256 object category dataset. 2007.

[App3] Jia Deng, Wei Dong, Richard Socher, Li-Jia Li, Kai Li, and Li Fei-Fei. Imagenet: A large-scale hierarchical image database. In *2009 IEEE conference on computer vision and pattern recognition*, pages 248–255. Ieee, 2009.

[App4] Xavier Glorot and Yoshua Bengio. Understanding the difficulty of training deep feedforward neural networks. In *Proceedings of the thirteenth international conference on artificial intelligence and statistics*, pages 249–256. JMLR Workshop and Conference Proceedings, 2010.

[App5] Kaiming He, Xiangyu Zhang, Shaoqing Ren, and Jian Sun. Delving deep into rectifiers: Surpassing human-level performance on imagenet classification. In *Proceedings of the IEEE international conference on computer vision*, pages 1026–1034, 2015.

[App6] Hugo Touvron, Matthieu Cord, Matthijs Douze, Francisco Massa, Alexandre Sablayrolles, and Hervé Jégou. Training data-efficient image transformers & distillation through attention. In *International conference on machine learning*, pages 10347–10357. PMLR, 2021.

[App7] Asish Bera, Zachary Wharton, Yonghuai Liu, Nik Bessis, and Ardhendu Behera. Attend and guide (ag-net): A keypoints-driven attention-based deep network for image recognition. *IEEE Transactions on Image Processing*, 30:3691–3704, 2021.

[App8] Alex Krizhevsky, Ilya Sutskever, and Geoffrey E Hinton. Imagenet classification with deep convolutional neural networks. *Advances in neural information processing systems*, 25, 2012.

[App9] Karen Simonyan and Andrew Zisserman. Very deep convolutional networks for large-scale image recognition. *arXiv preprint arXiv:1409.1556*, 2014.

[App10] Lucas Liebenwein, Alaa Maalouf, Dan Feldman, and Daniela Rus. Compressing neural networks: Towards determining the optimal layer-wise decomposition. *Advances in Neural Information Processing Systems*, 34:5328–5344, 2021.

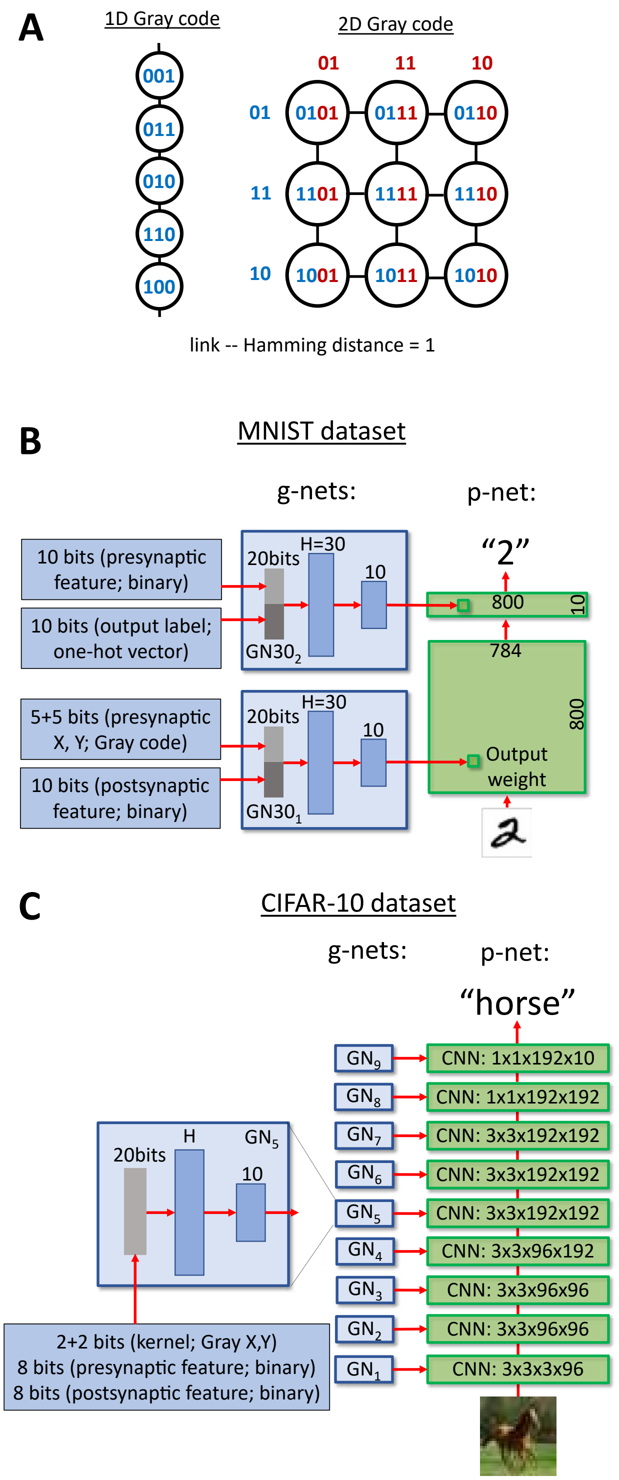

Figure S.1: Networks' structure for the MNIST and CIFAR-10 datasets (A) Neuron labels are assigned by a 2D Gray code so that neurons close in image space have similar (Hamming distance = 1) labels. (B-C) The structure of g-networks for the MNIST (B) and CIFAR-10 (C) datasets.

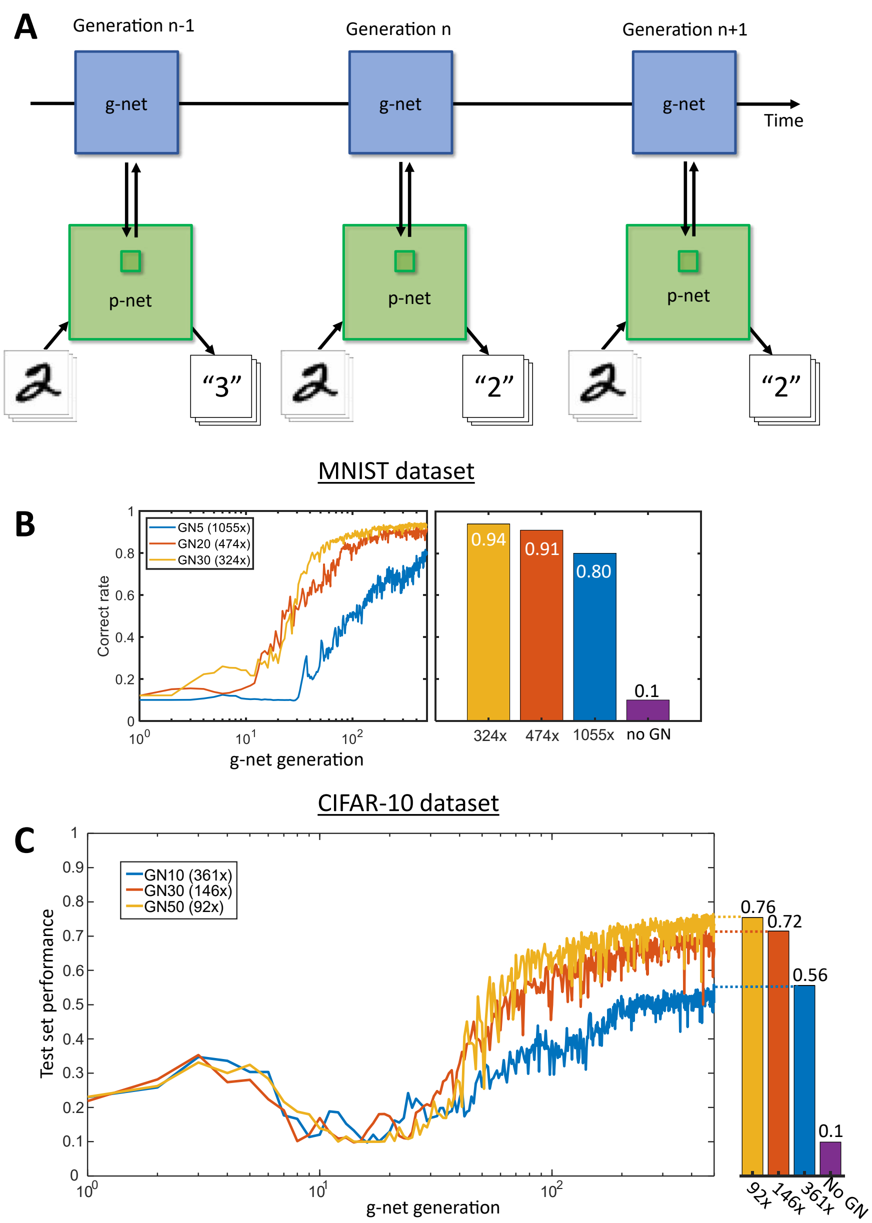

Figure S.2: Training strategies of g-networks. (A) The intermittent training strategy. G-network of generation n-1 is used to generate the p-network (down arrow, generation n). The p-network is trained using several minibatches without backpropagation of the gradients into the g-network. Then, the g-network is trained to match the adjusted p-network (up arrow in generation n). The resulting g-network in generation n is used to generate the p-networks in the next step (n+1). (B) The dynamics of training of g-network for the MNIST dataset. (C) The dynamics of training for the CIFAR-10 dataset. The small bump in performance at generations 2-7 is due to the annealing strategy used to initialize g-networks in this case [Eq. 3].

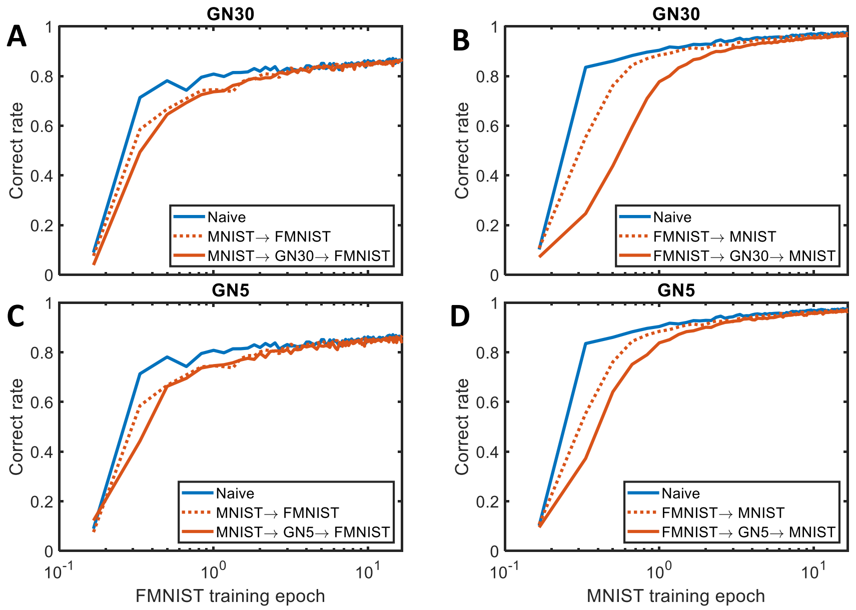

Figure S.3: The results of weight transfer between the MNIST and F-MNIST datasets compared to training networks based on random initialization (blue). Weight transfers using the entire set of weight matrices (dotted red lines) are contrasted to the transfer using g-networks (solid red). Two types of g-networks are used, with low (A, B) and high (C, D) compression. Transfers from the MNIST to F-MNIST (A, C) and from the F-MNIST to MNIST (B, D) show qualitatively the same results – training from scratch is better for this pair of datasets, even if the transfer occurs using the entire weight matrix (dotted, no compression).

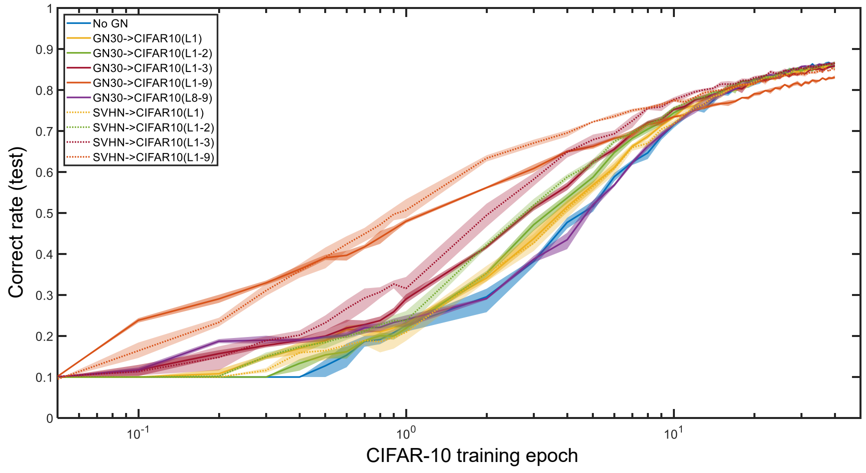

Figure S.4: The results of reverse transfer experiments from the SVHN to CIFAR-10 dataset. Training of a naïve weight matrix from scratch (blue) is contrasted with direct weight transfer (dotted lines) and transfer using the g-network (solid lines, GN30). Different sets of layers were transferred as indicated by the color map. Overall, these results show that, although different scenarios of weight transfer yield faster training than training from scratch (blue), the difference between uncompressed (dotted) and g-network compressed (solid) cases is not significant.

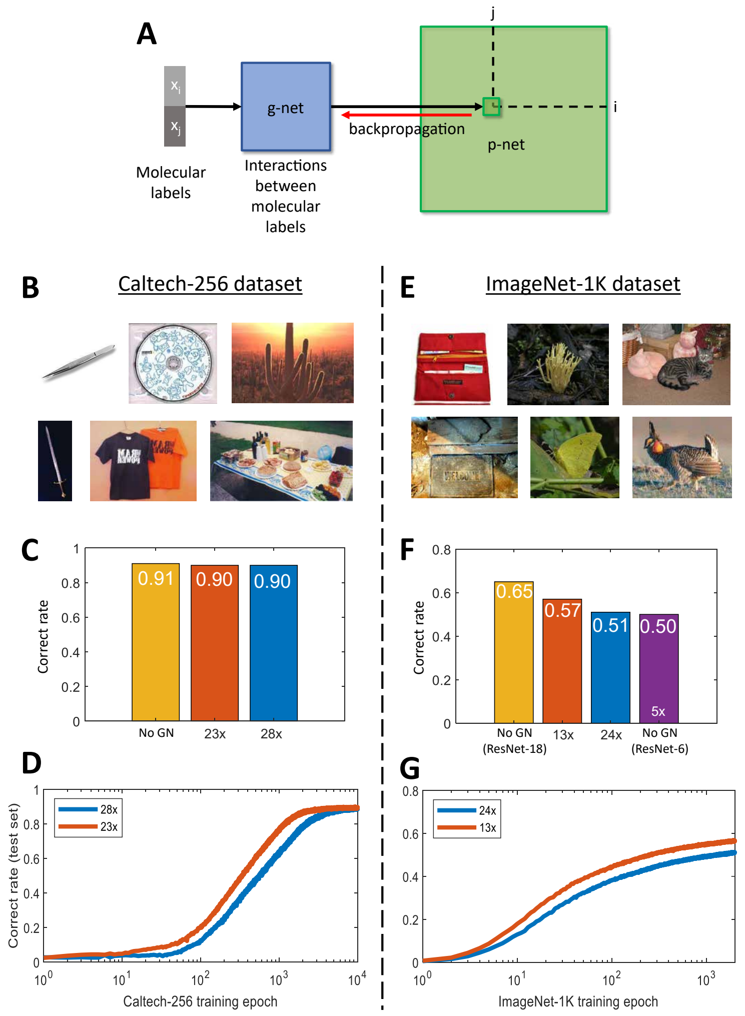

Figure S.5: (A) End-to-end backpropagation of errors from the output of the p-network to g-networks. (B-G) Applying the genomic bottleneck framework to the ResNet-18 model on (B-D) Caltech-256 and (E-G) ImageNet-1K datasets. (B) Examples of images in the Caltech-256 dataset. (C) Top-1 accuracy at some levels of compression. (D) Learning dynamics at 28-fold and 23-fold compression. (E) Examples of images in the ImageNet-1K dataset. (F) Top-1 accuracy at some levels of compression. (G) Learning dynamics at 24-fold and 13-fold compression.
